# A Rare Case of Suppurative Aspergillosis of the Thyroid

**DOI:** 10.1155/2013/956236

**Published:** 2013-09-12

**Authors:** Nikhil Dinaker Thada, Sampath Chandra Prasad, Bhasker Alva, Monika Pokharel, Kishore Chandra Prasad

**Affiliations:** Department of Otolaryngology Head & Neck Surgery, Kasturba Medical College, Manipal University, 5-7-712/3 ASRP Street, Dongerikeri, Kodialbail, Mangalore 575003, India

## Abstract

Suppurative fungal infection of the thyroid is an extremely rare condition even more so in an immunocompetent patient. Fungal infections of the thyroid usually occur in immunocompromised patients with hematological malignancies, recipients of bone marrow and solid organ allografts on immunosuppression, and patients with AIDS. A 65-year-old male presented with swelling in the front of the neck for 2 years. Examination revealed a 4 × 4 cm non-tender, firm swelling of the right lobe of the thyroid. The patient was taken up for a subtotal thyroidectomy. Intra-operatively, an abscess cavity with pus was found in the right lobe of the thyroid. Histopathology revealed features of fungal abscess and staining demonstrated fungal hyphae characteristic of *Aspergillus* ssp.

## 1. Introduction

Thyroid abscess was a common condition in the era before antibiotics. In the current medical environment, however, it is a clinical entity that is seldom encountered. Review of the published causes of thyroid abscess since 1980 demonstrated that although Gram-positive bacteria (*Staphylococcus* and *Streptococcus* species) remain the most common causes, there has been a marked decrease in the number of cases caused by mycobacteria, *Salmonella* species, and anaerobes when compared with the early part of the 20th century [1]. However, thyroid infections and even more so suppurative infections are very rarely caused by mycoses. The thyroid is resistant to microbial invasion because of its rich blood supply, iodine content, and capsule [2]. Although several fungi may infect the thyroid [3], thyroid fungal infection occurs rarely and is clinically overt in a minority of patients [2, 4, 5]. Of 415 previously reported cases of infectious thyroiditis (1900–1997), only 50 (12%) were fungal [2, 4, 5]. Since then only 12 cases have been reported till date. Fungal thyroiditis has been reported in patients with immunocompromised states or in the setting of disseminated disease. This case report deals with abscess of the thyroid caused by *Aspergillus *spp. in a patient who presented with a lump in the front of his neck with a competent immune system. 

## 2. Case Report

**Figure 1:**
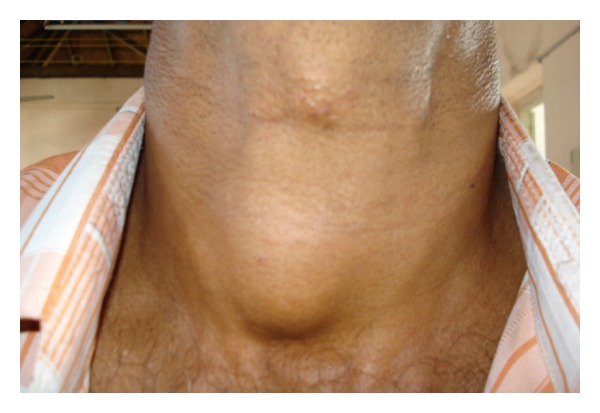
Preoperative photo showing the swelling in front of the neck.

**Figure 2:**
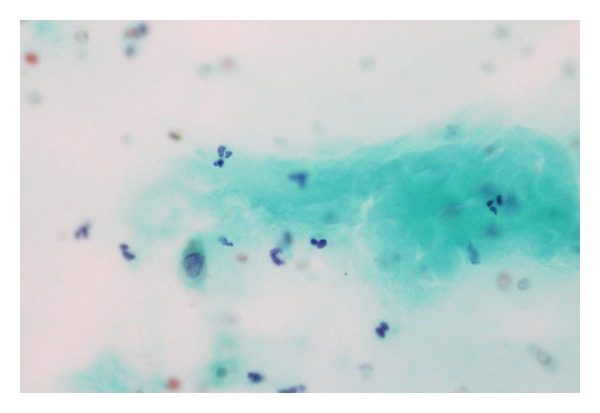
FNAC showing colloid.

**Figure 3:**
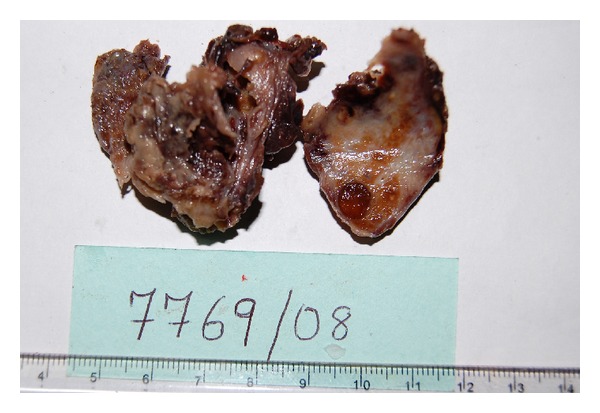
HPE specimen showing the abscess cavity in the right lobe of the thyroid.

**Figure 4:**
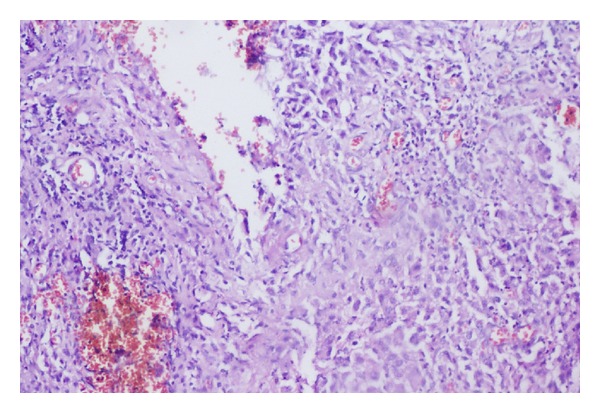
HPE photograph (×100 magnification) showing wall of abscess with granulation tissue and inflammatory infiltrate.

**Figure 5:**
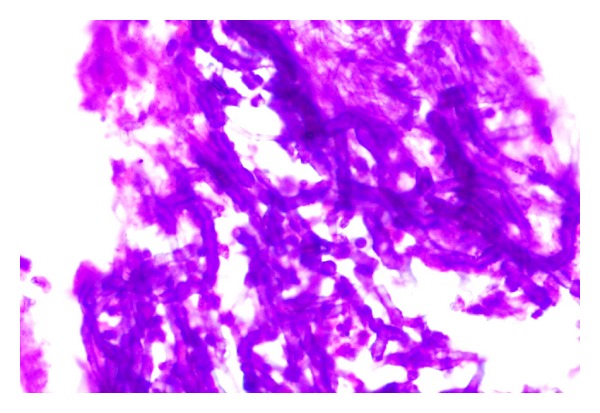
Hematoxylin and eosin (×400 magnification) HPE picture showing tufts of fungal hyphae.

**Figure 6:**
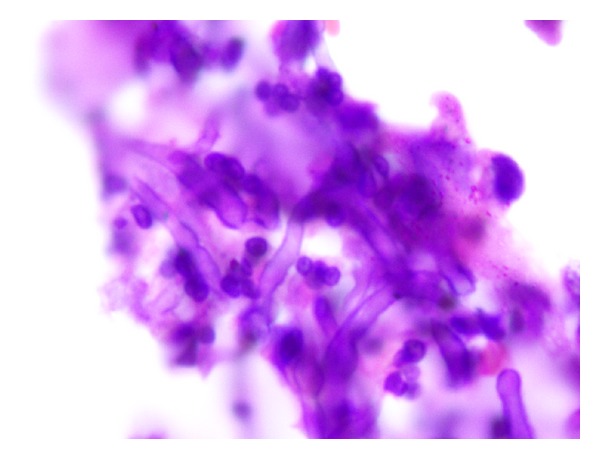
Periodic acid Schiff stained HPE picture showing 45 degrees branching of fungal hyphae characteristic of *Aspergillus* spp.

A 65-years-old male diabetic patient presented with swelling in the front of the neck for 2 years (Figure 1). There was no history of recurrent fever or change in voice. There was no history of cough or hemoptysis. There was no history suggestive of hypo- or hyperthyroidism. On examination, the patient was afebrile, normotensive, moderately built, and nourished. Local examination revealed a 4 × 4 centimeter nontender, firm swelling of the right lobe of the thyroid gland with nodular surface, extending laterally up to the anterior border of sternocleidomastoid muscle, superiorly 2 cm below the lower border of the thyroid cartilage, inferiorly up to the suprasternal notch. The swelling moved with deglutition and the overlying skin was normal. There was no thyroid bruit on auscultation. There were no palpable neck nodes. Indirect laryngoscopy finding was normal. The systemic and eye examinations were normal. Routine blood profile and urinalysis was within normal limits. Viral markers for HIV and HBsAg were negative. A random blood sugar (RBS) was valued at 264 mg/dL. The thyroid function tests were within the normal range. Electrocardiogram (ECG) and chest X-ray were within normal limits. Ultrasonogram showed heterogenous echotexture of the thyroid gland with multiple cystic areas within with internal echoes with one prominent hypoechoic mass lesion in the right lobe having irregular borders suggestive of an abscess. FNAC showed features suggestive of colloid goiter (Figure 2) with pus cells isolated from the abscess in the right lobe. After stabilizing the blood sugar levels, the patient was taken up for a subtotal thyroidectomy. Intraoperatively, an abscess cavity with pus was found in the right lobe of the thyroid. There was one 1 × 1 centimeter soft lymph node in the right trachea-esophageal groove. The histopathological examination (HPE) and staining of the specimen with periodic acid Schiff (PAS) of the postoperative specimen revealed features of fungal abscess involving the right lobe of the thyroid gland and adjacent soft tissue with associated fibrosis atrophy of thyroid tissue (Figures 3 and 4). Both hematoxylin and eosin and PAS stain demonstrated tufts of fungal hyphae with 45 degrees branching characteristic of *Aspergillus* ssp. (Figures 5 and 6). The patient was started on intravenous (IV) Cefotaxime, 1 gram 12th hourly, IV Metronidazole 500 milligram 8th hourly, and Amphotericin B 50 milligram in 5% dextrose 500 mL over 4–6 hours daily for 7 days. The patient developed chills and rigors after Amphotericin B infusion following which antifungal treatment was switched over to Tab Fluconazole at a dose of 200 mg BD for 7 days. RFT was repeated every 2nd day and showed a rise from 0.9 to 2.0 on the 6th day and 2.6 on the 10th day of starting antifungal treatment. The remainder of the postoperative days was uneventful. The patient was discharged on the 23rd postoperative day after 14 days of antifungal treatment. He was advised to continue antifungal treatment for a further 1 month. The patient is on followup and is free of the disease after 6 months. 

## 3. Discussion

Fungal infection of the thyroid is extremely uncommon. The ability of the thyroid gland to resist infection is well known and infection in the thyroid gland is rare, particularly so with the advent of widespread usage of antibiotics. The remarkable resistance of the thyroid gland to infection is attributed to many factors. A prosperous lymphatic and vascular supply, well-developed capsule, and high iodine content of the gland are various mechanisms suggested to account for this relative resistance to infection [6]. The pathogenesis of fungal thyroiditis entails hematogenous or lymphatic spread [2]. Although most cases of thyroid fungal involvement occur during dissemination in immunosuppressed patients, isolated thyroid histoplasmosis, coccidioidomycosis, and pneumocystosis have been reported. *Aspergillus *spp. are the predominant causative fungus for thyroiditis and asymptomatic thyroid infiltration [3]. The literature contains 21 case reports of overt thyroiditis caused by *Aspergillus *spp., with thyroid infiltration found postmortem in 10% to 35% of aspergillosis cases [3]. In contrast, thyroid infiltration is less common in other mycoses. Unsurprisingly, *P. jiroveci *is the most common cause of fungal thyroiditis in patients with AIDS, reflecting the high incidence of pneumocystosis in these patients [3]. Fungal thyroiditis typically begins with a brief hyperthyroid phase in which glandular destruction causes thyroid hormone release. Transient euthyroidism ensues, followed by hypothyroidism and, ultimately, recovery to euthyroidism. Hence, symptoms and laboratory evidence can range from those characteristics of hyperthyroidism (or even frank thyrotoxicosis) to those typical of hypothyroidism from fungal involvement, depending on the phase in which the condition is diagnosed [2]. Hyperthyroidism is more common with aspergillosis and coccidioidal infections, whereas hypothyroidism is more common with thyroiditis caused by *P. jiroveci.* Whether this pattern reflects the degree of glandular dysregulation by the aforementioned fungi or results from differences in the timing of diagnosis remains unknown. Patients with acute severe fungal infections may develop euthyroid sick syndrome, characterized by decreased levels of triiodothyronine and increased levels of normal thyroxine and thyrotropin-releasing hormone, as described in cases of paracoccidioidomycosis [3]. Fungal thyroiditis was diagnosed at autopsy as part of disseminated infection in a substantial number of patients without clinical manifestations and laboratory evidence of thyroid dysfunction [7].

Thyroid fungal involvement can manifest as subacute thyroiditis with local and systemic symptoms. Neck swelling and tenderness are common because of thyroid enlargement and dysphagia from esophageal compression; in severe cases, fatal respiratory failure from tracheal obstruction has occurred [3]. Clinically, local signs and symptoms of fungal thyroiditis are indistinguishable from other infectious thyroiditis which included fever, anterior cervical pain, thyroid enlargement sometimes associated with dysphagia [8], and dysphonia. Laboratory features of transient hyperthyroidism due to the release of thyroid hormone from follicular cell damage followed by residual hypothyroidism are described [6]. Many fungal thyroiditis have been reported to present as an abscess in the thyroid, though it is difficult to clinically determine this. The clinical presentation in our patient was similar to that of routine benign lesions of the thyroid. Clinically, there was no evidence of an infective pathology of the thyroid. 

Diagnosis of fungal thyroiditis is made by direct microscopy and culture of a fine needle aspirate and/or biopsy in most cases [4]. Fungal thyroiditis is diagnosed at autopsy as part of disseminated infection in a substantial number of patients without clinical manifestations and laboratory evidence of thyroid dysfunction. In our patient, the FNAC suggested colloid goiter with pus cells isolated from the abscess. Though the ultrasound picked up features of an abscess in the thyroid, the diagnosis of fungal thyroid abscess was only made postoperatively after HPE. In hindsight, the one thing we could have done was to send the aspirate for culture to clinch a diagnosis before taking the patient for surgery. 

Reported modes of management of thyroid abscess vary, but drainage remains an integral component of therapy for resolution of the infection [1]. If a diagnosis of suppurative fungal thyroiditis is made, the treatment usually involves aggressive surgical management, in addition to systemic antifungal therapy. Fungal thyroiditis without suppuration has been treated successfully with antifungals including Amphotericin, Fluconazole, and Itraconazole. Reduction of immunosuppression may prove helpful in disseminated disease. In preclinical studies, imidazoles, such as ketoconazole, have had antithyroid effects because of interference with iodine and thyroid peroxidase [9]. Nevertheless, administration of 200 to 600 mg/d of ketoconazole for 1 month did not affect thyroid function in euthyroid, hyperthyroid, or hypothyroid patients [3]. However, high-dose ketoconazole (1200 mg/d) may cause hypothyroidism [3].

## 4. Conclusion

Fungal thyroiditis is a rare condition but should be kept in mind in cases of immunocompromised conditions and disseminated disease. It is not uncommon for fungal thyroid infections to present as abscess which cannot be clinically distinguished from other benign lesions of the thyroid. Our case underlines the need for all cases of infective thyroiditis to be sent for both bacterial and fungal cultures, especially in the wake of pandemics of immunocompromised states like AIDS. Treatment of fungal thyroid abscesses usually involves aggressive surgical management, in addition to systemic antifungal therapy.

## 5. Summary

1. 62 cases of fungal thyroiditis were reported in the last 110 years.
2. Fungal thyroiditis has been reported in patients with immunocompromised states or in the setting of disseminated disease.
3. Our patient developed a fungal thyroid abscess despite a healthy immunity which is an extremely rare presentation
4. Fungal thyroiditis has been reported to present as an abscess in the thyroid, though it is difficult to clinically determine this.
5. In the wake of pandemics of immunocompromised diseases like AIDS, all cases of infective thyroiditis should be sent for both bacterial and fungal cultures.
